# CdS/CdSe Co-sensitized Solar Cells Based on Hierarchically Structured SnO_2_/TiO_2_ Hybrid Films

**DOI:** 10.1186/s11671-016-1493-7

**Published:** 2016-06-14

**Authors:** Zeng Chen, Chaochao Wei, Shengjun Li, Chunli Diao, Wei Li, Wenping Kong, Zhenlong Zhang, Weifeng Zhang

**Affiliations:** Key Laboratory of Photovoltaic Materials of Henan Province and School of Physics and Electronics, Henan University, Kaifeng, 475001 People’s Republic of China

**Keywords:** SnO_2_ nanosheet, SnO_2_/TiO_2_ hybrid films, Quantum dots, CdS, CdSe

## Abstract

SnO_2_ nanosheet-structured films were prepared on a fluorine-doped tin oxide (FTO) substrate using ZnO nanosheet as template. The as-prepared SnO_2_ nanosheets contained plenty of nano-voids and were generally vertical to the substrate. TiO_2_ nanoparticles were homogeneously deposited into the intervals between the SnO_2_ nanosheets to prepare a hierarchically structured SnO_2_/TiO_2_ hybrid film. The hybrid films were co-sensitized with CdS and CdSe quantum dots. The sensitized solar cells assembled with the SnO_2_/TiO_2_ hybrid film showed much higher photoelectricity conversion efficiency than the cells assembled with pure TiO_2_ films. The lifetime of photoinduced electron was also investigated through electrochemical impedance spectroscopy, which showed that the SnO_2_/TiO_2_ hybrid film electrode is as long as the TiO_2_ film electrode.

## Background

In recent years, quantum dot (QD)-sensitized solar cells have attracted remarkable attention because of the multiple exciton generation characters. The theoretical energy conversion efficiency of QD-sensitized solar cells (QDSCs) was calculated to be about 44.4 % which is much higher than that of the organic dye-sensitized solar cells [1]. Many narrow bandgap semiconductor QDs, such as PbS, CdS, and CdSe, have been extensively used to sensitize TiO_2_ photoanode [2–5]. Compared with TiO_2_, SnO_2_ has many advantages. Firstly, the energy gap of SnO_2_ is about 3.6 eV which may reduce the effect of UV light in the sunlight on the solar cell performance and improve their long-term stability [6]. Secondly, the electron mobility of SnO_2_ is about 150 cm^2^ V^−1^ s^−1^ which is much higher than that of TiO_2_ (1 cm^2^ V^−1^ s^−1^) [7, 8]. Thirdly, SnO_2_ films which are suitable for sensitized solar cells could be obtained without high temperature calcination [9, 10]. Therefore, some teams began to apply nanoporous SnO_2_ as photoanodes in QD-sensitized solar cells. Hossain et al. found that TiCl_4_ treatment can significantly increase the open circuit photovoltage of CdSe QD-sensitized SnO_2_ solar cells [11]. Then, they co-sensitized SnO_2_ films with CdS and CdSe QDs and obtained much higher short circuit current (*J*_SC_, 17.40 mA cm^−2^) than that of TiO_2_ film based QD-sensitized solar cells [12]. Cánovas et al. studied the electron transfer processes from PbSe quantum dots to SnO_2_ and found that the injection time of the photoexcited electron was vitally affected by the QD size [13]. Xiao et al. found that the shape of SnO_2_ might affect the photovoltage of SnO_2_-based QDSCs. They applied highly ordered SnO_2_ inverse opal films to QDSCs and obtained high open circuit voltage (*V*_OC_, 700 mV) and high short circuit current (10.13 mA cm^−2^). The total photoelectric transfer efficiency was about 4.37 % [14].

Specific nanostructure of nanoparticles, such as nanorod, nanosheet, and nanowire, could bring some distinctive properties. Some teams had attempted to prepare SnO_2_ nanosheets. Li Y et al. synthesized SnO_2_ nanosheets by hydrothermal method from SnCl_2_ and NaOH in ethanol/water solution [15]. Fei L et al. prepared SnO_2_ nanosheets using graphite sheets as template [16]. Dong CJ et al. obtained Pt-functionalized SnO_2_ nanosheets by a facial solution combustion method [17].

**Fig. 1:**
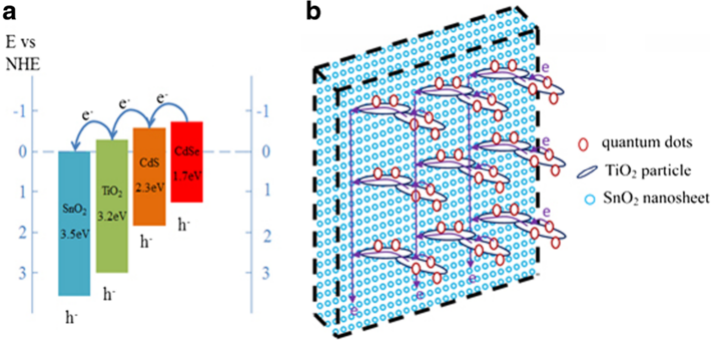
Diagrammatic sketch of band energy **(a)** and structure **(b)** of SnO_2_/TiO_2_ hybrid film

In this experiment, we prepared SnO_2_ nanosheet-structured films using ZnO nanosheet as template. The as-prepared SnO_2_ nanosheets contain plenty of nano-voids and are generally vertical to the substrate, which should provide an efficient collection path for the photoinduced electron. To obtain SnO_2_/TiO_2_ composite films, TiO_2_ nanoparticles were deposited on SnO_2_ nanosheet through electrophoresis method. And these films were introduced into QDSCs. From the band energy structure of SnO_2_ and TiO_2_, we can see that the electron can transfer from the conduction band of TiO_2_ to that of SnO_2_ shown in Fig. 1a. So the SnO_2_/TiO_2_ composite films could combine the advantages of both SnO_2_ nanosheet and TiO_2_ particle. The photoexcited charges were separated efficiently on the surface of TiO_2_ nanoparticles. Thereafter, photoinduced electron will be collected by SnO_2_ nanosheets and transported to the fluorine-doped tin oxide (FTO) substrate fluently. The schematic diagram of these processes is shown in Fig. 1b.

## Methods

### Materials

Zinc nitrate hexahydrate (Zn(NO_3_)_2_·6H_2_O), zinc acetate (Zn(CH_2_COO)_2_), ammonium hexafluorostannate ((NH_4_)_2_SnF_6_), boric acid (H_3_BO_3_), cadmium acetate (Cd(CH_2_COO)_2_), selenium powder (Se), sodium thiosulfate (Na_2_S_2_O_3_.5H_2_O), nitrilotriacetic acid trisodium salt (C_6_H_8_NNa_3_O_7_), copper nitrate trihydrate (Cu(NO_3_)_2_·3H_2_O), and sodium sulfide (Na_2_S) were all purchased from Sinopharm Chemical Reagent Co. (SCRC, China). Ethanol and methanol were purchased from Aladdin Reagent Co. (China) with a purity >99.9 %. All of these materials were used as received without any further purification.

The electrodeposition of ZnO nanosheets was carried out in a simple three-electrode glass cell. The precursor solution (for ZnO deposition) consisted of 0.05 M Zn(NO_3_)_2_·6H_2_O and 0.1 M KCl. The working electrode was FTO glass substrates (10 × 10 mm). The reference electrode was Ag/AgCl electrode with saturation potassium chloride aqueous solution, and the counter electrode was Pt metal sheet. The distance between the working electrode and the counter electrode was about 3.5 cm. The deposition temperature was fixed at 70 °C by an oil bath. The deposition potential was controlled to be −1.1 V. The deposition time was controlled to be 30 min unless specially instructed. The deposited samples were cleaned with deionized water, dried at room temperature, and annealed at 450 °C for 30 min in the air atmosphere. The deposition time was controlled to be 30 min. For the formation of SnO_2_ layer, the ZnO nanosheets were then immersed in a mixture of 3 mL 0.15 mol L^−1^ (NH_4_)_2_SnF_6_, 1 mL 0.5 mol L^−1^ H_3_BO_3_, and 1 mL deionized water [18]. The immersion time was 4 h to convert all ZnO nanosheets to SnO_2_ nanosheets. The SnO_2_ nanosheets were cleaned with deionized water, dried at room temperature, and sintered at 500 °C for 30 min under air atmosphere. Then, the SnO_2_ nanosheet-structured films were immersed into 40 mM TiCl_4_ aqueous solution at 70 °C. The immersion time was controlled to be 40 min. The TiCl_4_-treated SnO_2_ films were annealed at 500 °C for 30 min under air atmosphere. Then, commercial TiO_2_ nanoparticles (P25) were deposited on the SnO_2_ nanosheet through electrophoresis method in a colloid solution (0.5 g P25 dispersed in a mixture of 8 mL butanol, 4 mL isopropanol, and 2 mL ethanol). In the electrophoresis processes, an FTO glass (1 × 2 cm^2^, 15 Ω sq^−1^; OPV Tech) was used as the cathode and another FTO glass was used as the anode. The distance between the two electrodes was maintained at 1 cm, and the DC power supply was set at 48 V. The electrophoresis time is 10 s. The SnO_2_/TiO_2_ hybrid films were sintered at 500 °C for 30 min.

### CdS/CdSe Co-sensitized Photoanodes and Solar Cell Device Fabrication

CdS and CdSe quantum dots were deposited on these nanoporous films (pure SnO_2_ film, TiCl_4_-treated SnO_2_ film, SnO_2_/TiO_2_ hybrid film, or pure TiO_2_ film) in sequence. The deposition process was summarized as follows. Firstly, the nanoporous films were sensitized with CdS quantum dots by successive ionic layer adsorption and reaction (SILAR) method. The deposition process was summarized as follows: (i) The pure ZnO and ZnO/TiO_2_ composite samples were firstly dipped in the 0.1 M Cd(NO_3_)_2_ ethanol solution for 1 min, then rinsed with ethanol for 1 min, followed by dipping in the 0.1 M methanol solution for 1 min and then rinsing with methanol for 1 min. (ii) The former processes were repeated 14 times in order to grow sufficient amount of CdS QDs on the films. Secondly, the CdS-sensitized films were immersed in a mixture of aqueous solution, 0.2 mol L^−1^ Na_2_SeSO_3_, 0.16 mol L^−1^ C_6_H_8_NNa_3_O_7_(NTA-3Na), and 0.08 mol L^−1^ Cd(CH_2_COO)_2_ (V:V:V = 1:1:1), for 4 h. Thirdly, the CdS/CdSe co-sensitized films were passivated with ZnS by immersion into 0.1 mol L^−1^ Zn(CH_2_COO)_2_ and 0.1 mol L^−1^ Na_2_S aqueous solution in sequence. For QDSCs fabrication, CuS counter electrodes were prepared according to the reported literature [19]. The polysulfide aqueous solution of 1 mol L^−1^ Na_2_S, 1 mol L^−1^ S, and 0.1 mol L^−1^ NaOH was used as the QDSCs electrolyte.

### Measurement and Characterization

The crystalline phase of the samples was characterized by DX-2700 X-ray diffractometer (XRD) with a monochromatized CuK irradiation (*k* = 0.154145 nm). The morphology was studied using JSM-7001F field emission scanning electron microscope (FE-SEM). Energy dispersive spectroscopy analysis (EDS) was obtained from Bruker-ASX (Model Quan-Tax 200).

The assembled QDSCs were tested under simulated sunlight (AM 1.5G illumination) from a Newport Oriel Solar Simulator (model 94043A, Oriel) using Keithley 2440 Source Meter. The light intensity was calibrated with a standard Si solar cell provided by Newport Oriel. The active cell area of the testing QDSCs was 0.25 cm^2^. The monochromatic incident photon-to-electron conversion efficiency (IPCE) was measured using an IPCE system (QS 500ADX, Crowntech, Inc.). The testing ranged from 300 to 800 nm. A 150-W tungsten halogen lamp was used as the light source to generate a monochromatic beam. A silicon solar cell was used as the reference during the IPCE measurement. An electrochemistry workstation (IM6) was used to investigate the electrochemical impedance spectra (EIS) of QDSCs. This measurement was also carried out with the same structured QDSCs as that used in the former experiments. The impedance measurement of QDSCs was recorded under dark condition at the bias potential of −0.6 V over a frequency range of 0.1–1 MHz with an AC amplitude of 10 mV.

## Results and Discussion

ZnO nanosheet-structured film was firstly electrodeposited on FTO substrate. Then, the ZnO nanosheet-structured film was immersed in (NH_4_)_2_SnF_6_ aqueous solution. The ${\mathrm{SnF}}_6^{2-}$ ions in the solution will hydrolyze and form SnO_2_ nanoparticles on the surface of ZnO nanosheets following Eq. 1. The generated F^−^ ion in Eq. 1 could be trapped by boric acid as described in Eq. 2. The H^+^ in HBF_4_ would dissolve ZnO into the solution. If the immersion time was long enough, all the ZnO nanosheets on the FTO substrate might be totally dissolved into the solution. As a result, pure SnO_2_ nanoporous nanosheet film was prepared. The chemical reactions in the treatment process might proceed with the following mechanisms [18]:

1 $$ {\mathrm{SnF}}_6^{2-}+2{\mathrm{H}}_2\mathrm{O}\to {\mathrm{SnO}}_2+6{\mathrm{F}}^{-}+4{\mathrm{H}}^{+} $$

2 $$ {\mathrm{H}}_3{\mathrm{BO}}_3+4\mathrm{H}\mathrm{F}\to {\mathrm{H}\mathrm{BF}}_4+3{\mathrm{H}}_2\mathrm{O} $$

3 $$ \mathrm{Z}\mathrm{n}\mathrm{O}+2{\mathrm{H}}^{+}\to {\mathrm{Zn}}^{2+}+{\mathrm{H}}_2\mathrm{O}. $$

**Fig. 2:**
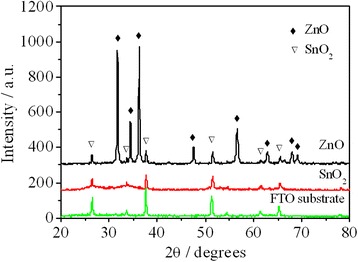
XRD of FTO substrate and the prepared pure ZnO and SnO_2_ films

Figure 2 shows the XRD patterns for the pure ZnO films before and after 4 h immersion in the (NH_4_)_2_SnF_6_ aqueous solution. Before the treatment of (NH_4_)_2_SnF_6_ aqueous solution, there is a series of narrow peaks at 31.76°, 34.4°, 36.24°, 47.56°, and 56.6° in the X-ray diffraction spectra. These peaks indicate the growth of wurtzite-structured ZnO (hexagonal phase, space group P63mc) (JCPDS database card no. 36-1451). Other peaks are all in accordance with the diffraction peaks of the FTO substrate. After 4 h of immersion in the (NH_4_)_2_SnF_6_ aqueous solution, no diffraction peaks of ZnO can be found in the spectrum which indicates that all ZnO nanosheets have been dissolved into the solution. The ultimate sample consists of pure SnO_2_.

**Fig. 3:**
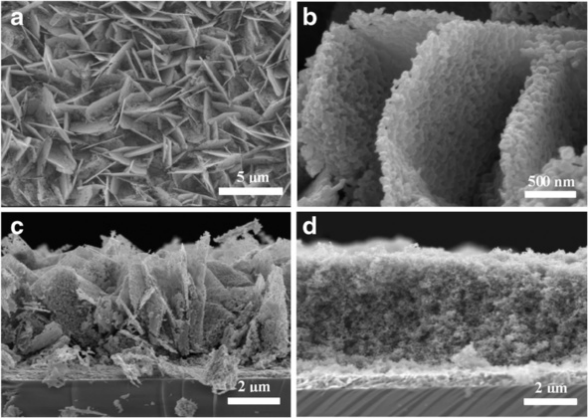
SEM of the prepared films. **a**, **b** Top view of pure SnO_2_ film. **c** Cross section of pure SnO_2_ film. **d** Cross section of SnO_2_/TiO_2_ hybrid film

Figure 3a–c shows the top view and cross section of the prepared pure SnO_2_ nanosheet film. The as-prepared SnO_2_ films are not as regular as ZnO films, but it maintains the nanosheet structure. And the SnO_2_ sheets are also generally vertical to the substrate. The microstructure of the SnO_2_ nanosheet is much different from that of ZnO. There are plenty of homogeneous nano-voids distributed between SnO_2_ nanoparticles. These nano-voids are suitable for the deposition of TiO_2_ nanoparticles and quantum dots. The thickness of the film is about 6 μm.

The conduction band edge of SnO_2_ is 0.4 V (versus the standard hydrogen electrode (SHE)) which is more positive than that of TiO_2_. Photoexcited electrons in the conduction band of SnO_2_ undergo serious back reactions [20]. Coating SnO_2_ with thin layers of TiO_2_ is an efficient way to inhibit these back reactions. So the as-prepared SnO_2_ sheet films were treated in 40 mM TiCl_4_ aqueous solution at 70 °C for 40 min for the covering of a passivation layer of TiO_2_. Then, commercial TiO_2_ nanoparticles (P25) were deposited on the SnO_2_ sheets through electrophoretic method. Figure 3d shows the cross section of the TiO_2_ nanoparticle-covered SnO_2_ film. It can be seen that TiO_2_ nanoparticles were homogeneously filled in the intervals between SnO_2_ nanosheets. From the cross section of SnO_2_/TiO_2_ hybrid film, the SnO_2_ nanosheets become so distinct that we can almost not found them. This change of SnO_2_ might be caused by the mild dissolution of SnO_2_ in TiCl_4_ treatment. And the TiO_2_ nanoparticles were efficiently coated on SnO_2_ skeleton.

**Fig. 4:**
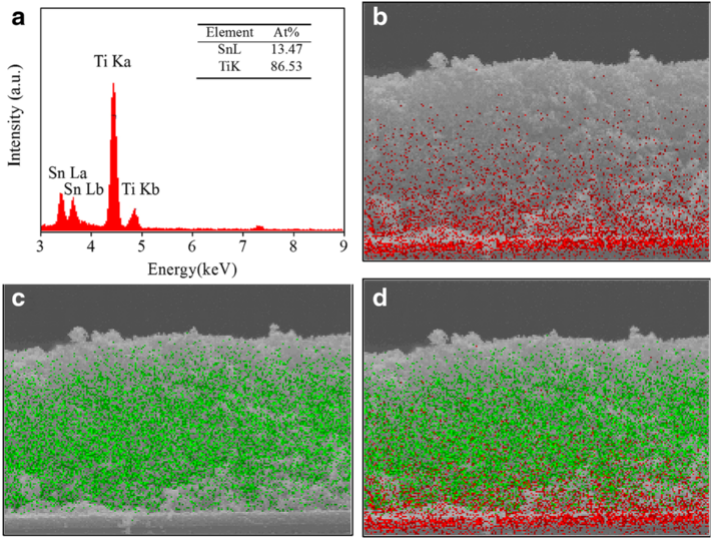
EDS of the prepared films. **a** Sn and Ti content. **b** Sn element distribution map. **c** Ti element distribution map. **d** Combination of Sn and Ti element distribution map

X-ray EDS was carried out to confirm the final composition of the SnO_2_/TiO_2_ hybrid film. The EDS spectra are shown in Fig. 4. The peaks at about 3.4, 3.6, 4.44, and 4.82 KeV should correspond to Sn(La), Sn(Lb), Ti(Ka), and Ti(Kb), respectively. The composition analysis revealed that the ratio between Sn and Ti was about 13.5:86.5. The element distribution diagrams are also given in Fig. 4b–d. It can be seen that Sn element exists throughout the whole films. At the bottom of the film, there is a gathering of Sn element which should be attributed to the F-coated SnO_2_ layer on the glass substrate. The Ti element was homogeneously filled in the SnO_2_ frameworks.

**Fig. 5:**
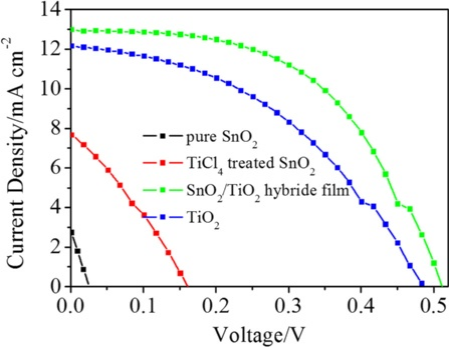
*J*-*V* characteristic of the prepared CdS/CdSe co-sensitized QDSCs

To investigate the effects of TiCl_4_ treatment and coverage of TiO_2_ nanoparticles on the photovoltaic characteristics of the SnO_2_ films, these nanoporous films (pure SnO_2_ film, TiCl_4_-treated SnO_2_ film, SnO_2_/TiO_2_ hybrid film, or pure TiO_2_ nanoparticle film) were all co-sensitized with CdS and CdSe quantum dots. They were assembled with CuS counter electrodes, separately, to form a complete QDSC. The *J*-*V* curves of the former assembled QDSCs are shown in Fig. 5. The pure SnO_2_ nanosheets film shows poor photovoltaic characteristics. After TiCl_4_ treatment, the *J*_SC_ and *V*_OC_ increased from 2.9 mA cm^−2^ and 25 mV to 7.7 mA cm^−2^ and 161 mV, respectively. These characteristic parameters are significantly improved because the recombination reaction at the surface of SnO_2_ nanosheet was restricted by the treatment of TiCl_4_. However, the TiO_2_ layer might not be enough to get rid of the back reaction on SnO_2_ nanosheet film. The reason for the low photocurrent might be attributed to the low quantity of QDs on the photoelectrode. The specific surface area is a major factor affecting the loading of QDs. Those are also the reasons why the characteristic parameters are lower than that of the former reports [11–13]. To solve this problem, commercial TiO_2_ nanoparticles (P25) were deposited on the SnO_2_ nanosheet films. From Fig. 4, it can be seen that the photoelectric conversion properties of the photoanode are significantly improved. The *J*_SC_, *V*_OC_, and fill factor (*FF*) are about 13.0 mA cm^−2^, 514 mV, and 52.2 %, respectively. The total photoelectric conversion efficiency (*η*) is about 3.49 %. There might be two reasons for the improvement of the photoelectric properties after the deposition of commercial TiO_2_ nanoparticles (P25). One is the significant improvement of the specific surface area, the film electrode. The other reason is that the deposition of commercial TiO_2_ nanoparticles (P25) further isolated SnO_2_ from QDs and electrolyte, which further restricted the recombination of the photoexcited electron in the SnO_2_ conductive band. As a reference, pure TiO_2_ nanoparticles were also directly deposited on the FTO substrate under the same electrophoretic time as that used in the former experiments. The photoelectric conversion efficiency is about 2.51 % which is much lower than that of QDSCs assembled with SnO_2_/TiO_2_ hybrid films. All the characteristic parameters are shown in Table 1.

**Table 1:** Detailed photovoltaic parameters of the QDSCs obtained from Fig. 5

Photoanodes	*V*_OC_ (mV)	*J*_SC_ (mA cm^−2^)	FFP (%)	Efficiency (%)
SnO_2_	25	2.9	23.8	0.02
SnO_2_ + TiCl_4_	161	7.7	29.7	0.37
SnO_2_ + TiCl_4_ + P25	514	13.0	52.2	3.49
P25	488	12.2	42.0	2.51

**Fig. 6:**
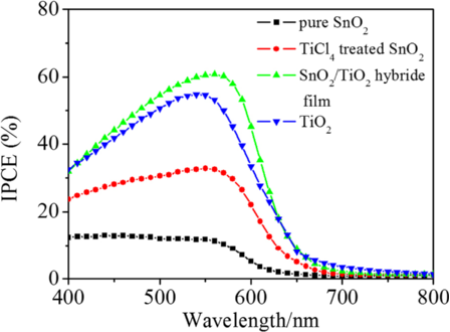
IPCE characteristic of the CdS/CdSe co-sensitized QDSCs

Figure 6 shows the IPCE spectra of QDSCs assembled with these different photoanodes. IPCE spectra reflect the light response of photovoltaic devices at different light wavelengths, which is directly related to photocurrent density and can be calculated from Eq. 4.

4 $$ \mathrm{IPCE}\ \left(\%\right)=1240{J}_{\mathrm{SC}}/\left(\lambda {P}_{\mathrm{in}}\right), $$

where *J*_SC_ is the short circuit photocurrent density at a single wavelength, *λ* is the wavelength of the incident light, and *P*_in_ is the power of the incident light. The light absorbed by the photoanodes ranged from 400 nm to about 700 nm which is in accordance with the absorption range of CdS and CdSe. Comparing the curves obtained by different photoanodes, it can be seen that TiCl_4_ treatment and TiO_2_ nanoparticle coverage dramatically enhanced the IPCE values during the 400–700 nm, which is in accordance with the results of *J*-*V* curves.

**Fig. 7:**
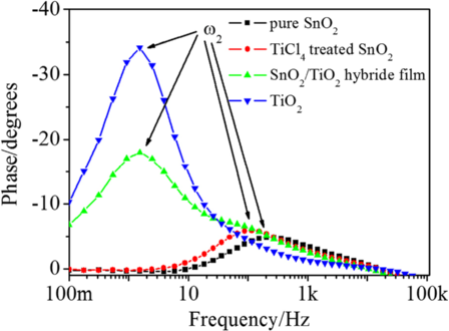
Bode phase plots of the CdS/CdSe co-sensitized photoanodes

Electrochemical impedance spectroscopy (EIS) is an efficient method to investigate the recombination process of the photoexcited electrons. EIS was carried out on SnO_2_, TiCl_4_-treated SnO_2_, SnO_2_/TiO_2_ hybrid film, and TiO_2_ film photo-electrodes under dark condition. A bias potential, −0.6 V, was applied in the testing process. Figure 7 shows the Bode phase plots of the QDSCs assembled with SnO_2_, TiCl_4_-treated SnO_2_, SnO_2_/TiO_2_ hybrid film, and TiO_2_ film. According to the previous work, there should be an electrochemical process (ω1) at high frequency (10^3^–10^5^ Hz) to correspond to the charge-transfer processes occurring at the counter electrode/electrolyte interface [21]. But it is not obvious in this experiment. However, there is an obvious electrochemical reaction process at the frequency range from about 1 to 10^3^ Hz which was marked as *ω*_2_. This process corresponds to the charge-transfer processes occurring at the SnO_2_ (TiCl_4_-treated SnO_2_ film, SnO_2_/TiO_2_ hybrid film, or P25 film)/electrolyte (or QD) interface [21]. The characteristic frequency of *ω*_2_ may reflect the electron lifetimes (*τ*_e_) of the injected electrons [22]. The lifetimes (*τ*_e_) of the photoexcited electron in the photoanodes were determined using the following equation (Eq. 5):

5 $$ {\tau}_e=\frac{1}{2\pi {f}_{\max }}. $$

The characteristic frequency of these photoanodes, SnO_2_, TiCl_4_-treated SnO_2_, SnO_2_/TiO_2_ hybrid film, and pure TiO_2_ film, were 202.7, 115.4, 1.5, and 1.5 Hz, respectively. According to Eq. 5, the electron lifetimes (*τ*_e_) were calculated to be about 0.8, 1.4, and 106.2 ms for the SnO_2_, TiCl_4_-treated SnO_2_, SnO_2_/TiO_2_ hybrid film, and TiO_2_ electrodes, respectively. It can be seen that TiCl_4_ treatment exactly inhibited the recombination reaction of SnO_2_ nanosheet electrode. But the effects of TiCl_4_ treatment are very finite. After the coverage of TiO_2_, the electron lifetime was lengthened by two orders to the same value as that of the pure TiO_2_ photoanode. It can be seen that the SnO_2_/TiO_2_ hybrid electrode might combine the advantages of both SnO_2_ nanosheet and TiO_2_ nanoparticle. This result is in accordance with the *J*-*V* curves.

## Conclusions

SnO_2_ nanosheet-structured films were prepared using ZnO nanosheet as template. The as-prepared SnO_2_ nanosheets contained plenty of nano-voids and were generally vertical to the substrate. TiO_2_ nanoparticles were homogeneously deposited into the intervals between SnO_2_ nanosheets to prepare hierarchically structured SnO_2_/TiO_2_ hybrid film. The hybrid films were co-sensitized with CdS and CdSe quantum dots. The photoinduced electron showed the same lifetimes in this SnO_2_/TiO_2_ hybrid film as that in the pure TiO_2_ particles films. But the SnO_2_/TiO_2_ hybrid film photoanode had higher IPCE than pure TiO_2_ nanoparticle photoanode. The total photoelectric conversion efficiency was about 3.49 %.
